# A real-world feasibility evaluation of LLM-based clinical prediction: emergency department return visit admission across two academic medical centers

**DOI:** 10.21203/rs.3.rs-9733941/v1

**Published:** 2026-06-02

**Authors:** Jinsong Liu, Katherine Brown, Michelle J. Ma, Arindam RoyChoudhury, Bradley A Malin, Allison McCoy, Adam Wright, Jessica S. Ancker, Tony Rosen, Jin Ho Han, Peter A D Steel, Yiye Zhang

**Affiliations:** Weill Cornell Medicine; Vanderbilt University Medical Center; Weill Cornell Medicine; Weill Cornell Medicine; Vanderbilt University Medical Center; Vanderbilt University Medical Center; Vanderbilt University Medical Center; Vanderbilt University Medical Center; Weill Cornell Medicine; Vanderbilt University Medical Center; Weill Cornell Medicine; Weill Cornell Medicine

## Abstract

Large language models (LLMs) are increasingly applied to clinical prediction tasks and offer significant promise for real-world impact. Whether that promise translates into deployment, however, depends on several feasibility constraints, performance, data requirements, operational cost, and safety, that are rarely evaluated together. Using emergency department (ED) return visit admission (RVA) prediction as a clinical use case, we developed and tested a two-stage LLM-based approach at two academic medical centers. In the first stage, an LLM generates structured severity summaries from ED documentation and structured EHR variables; in the second stage, these summaries inform RVA prediction via zero-shot or in-context learning (ICL). We evaluated the framework on four feasibility axes simultaneously: discrimination performance, data requirements, per-encounter token consumption, and hallucination risk in LLM-generated severity summaries. The LLM-based pipeline outperformed the strongest traditional ML baseline at WCM (Gemini ICL = 3 AUC 0.746 vs. CatBoost AUC 0.723; DeLong p = 0.044), but not at VUMC, where logistic regression on structured data alone (AUC 0.799) matched the best LLM configuration (GPT-5 mini AUC 0.789, n.s.). Absolute discrimination at VUMC was higher than at WCM for both approaches, indicating that the cross-site difference reflects local documentation and structured-data informativeness rather than LLM failure. We find that the deploy-or-not decision for an LLM-based clinical prediction pipeline is primarily a feasibility question first, rather than an architectural one. This decision is site-conditional, tied to how much of the predictive signal is already captured by structured EHR variables at a given site.

## Introduction

Clinical prediction with electronic health record (EHR) data increasingly poses a design choice between two paradigms: locally trained machine learning (ML) models built on structured variables, and large language models (LLMs) that integrate structured and narrative inputs into probabilistic outcome estimates^1–3^. LLMs have shown promise for risk prediction across heterogeneous clinical tasks^4^, including triage and severity assessment in the emergency department (ED)^5–10^, yet a parallel body of work has shown that LLMs can underperform locally trained ML on discrimination, calibration, fairness, and robustness when systematically benchmarked^11^. For practitioners weighing deployment, translating these mixed findings into actionable decisions is difficult, because most evaluations characterise a single dimension, typically discrimination, while real-world adoption is jointly constrained by performance, data requirements, operational cost, and safety^12,13^.

We examine this deployment question using ED return visit admission (RVA), an unplanned hospital admission within a defined window after ED discharge, as a clinically consequential and methodologically demanding case study. RVA exhibits the properties that make clinical prediction difficult: low prevalence (1–2% of ED discharges)^14–19^, substantial downstream risk including ICU admission, prolonged hospitalisation, and elevated in-hospital mortality among returning patients^20–23^, and an inherent reliance on clinician reasoning that is documented predominantly in narrative notes^24–26^. Existing RV and RVA prediction models trained on structured EHR data achieve only modest discrimination^27–32^, and approaches incorporating clinical text via traditional NLP^33–35^ have shown that narrative documentation carries predictive signal not captured by structured fields alone. Whether LLM-based pipelines improve on these methods, and whether any such gains justify their costs, remains unclear.

Direct head-to-head comparisons between LLM-based and traditional ML approaches for clinical prediction are uncommon, and those that exist typically evaluate a single performance dimension. Brown et al. reported sobering multidimensional results for GPT-3.5 and GPT-4 on clinical prediction tasks but did not quantify cost^11^. Williams et al. tested ChatGPT for ED disposition recommendations but did not benchmark against locally trained ML or measure operational overhead^10^. Multi-site LLM evaluations remain rare^36^, despite extensive evidence that ML clinical models degrade across institutions and even across sites within a network^37–42^. The economic feasibility dimension is particularly underexplored: although commercial LLM inference incurs per-encounter costs that scale linearly with patient volume, potentially prohibitive at ED throughput^13,39^, few studies report tokens consumed, dollars per prediction, or latency alongside discrimination. Hallucination risk in LLM-generated clinical content is similarly understudied as a deployment-relevant outcome.

We address this gap by evaluating a two-stage LLM-based framework for RVA prediction across two academic medical centres, Weill Cornell Medicine (WCM) and Vanderbilt University Medical Center (VUMC), against traditional ML baselines. In the first stage, an LLM produces a structured severity summary from each encounter's narrative documentation and structured EHR data, motivated by prior work showing that LLM-based abstraction outperforms raw text ingestion for ED prediction tasks^7^; in the second stage, the summary and structured variables drive prediction via zero-shot or in-context learning^43–46^, with probabilistic outputs derived from token-level log-probabilities^47^. Critically, we evaluate this framework on four feasibility axes simultaneously: (1) discrimination performance, (2) data requirements, (3) per-encounter operational cost across commercial APIs and open-source models, and (4) hallucination rate in LLM-generated severity summaries. Both commercial (Gemini 2.5 Flash at WCM, GPT-5 mini at VUMC) and open-source (Qwen3–8B at both sites) backbones are evaluated.

This study makes three contributions. We provide the first multi-site, head-to-head comparison of an LLM-based clinical prediction pipeline against locally-trained ML baselines for ED return visit admission prediction across 110,000 encounters at two academic medical centers. We evaluate the framework on four feasibility axes simultaneously, discrimination, data requirements, operational cost, and hallucination risk, establishing a reusable evaluation template for clinical LLM deployment decisions. We document a site-conditional LLM advantage, propose that its presence depends on the relative informativeness of structured versus narrative EHR data at a given site, and recommend site-specific structured-only baseline evaluation as a practical pre-deployment check.

## Results

We selected visits by adult patients who were discharged from an index visit to a WCM emergency department with records that included clinical notes. We excluded visits by patients with unknown gender (n = 167) to minimize the likelihood of missing data. From a total of 1,004,451 emergency department encounters between 2022-01-01 and 2025-08-30 in WCM, these criteria produced an analytic sample of 892,179 visits. A total of 576,839 ED visits at VUMC ED between 2022-01-01 and 2025-08-30 resulted in a final dataset of 153,751 ED visits. To reduce computational and time requirements for the analysis, we randomly sampled 100,000 WCM ED visits and 10,000 VUMC ED visits from this final dataset to form the study cohort. Among the WCM and VUMC sampled encounters, 1,247 visits (1.25%) and 106 visits (1.06%) resulted in a 9-day RVA, respectively. Table 1 describes the patient demographics in the study cohort with statistically significant differences between RVA cases and non-RVA cases across age, gender, race, and ethnicity at WCM and VUMC. Unlike at WCM, the only statistically significant differences between RVA cases and non-RVA cases are for age and race in VUMC.

**Figure 1:**
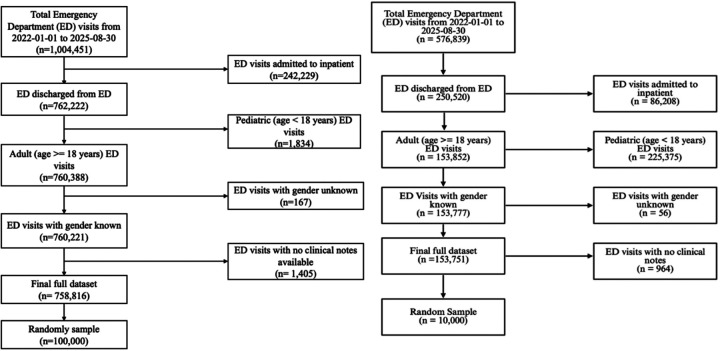
Cohort selection at WCM and VUMC

**Figure 2:**
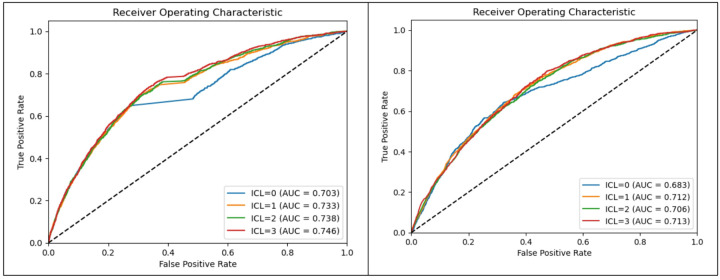
Performance of Gemini2.5-Flash (left) and Qwen3-8B (right) on WCM data

The full inclusion criteria are described in Figs. 1 and 2. Discrimination performance for the two-stage framework and all baselines is summarised in Table 2; full ablation results, including ICL sweeps, embedding-based variants, and subgroup analyses, are presented in Table 3.

### Discrimination performance

The LLM-based pipeline achieved the strongest discrimination at WCM but not at VUMC, where logistic regression on structured EHR data alone achieved comparable performance at substantially lower cost. Across both sites, absolute discrimination was higher at VUMC than at WCM for both LLM and ML approaches, indicating that the cross-site contrast reflects local data informativeness rather than failure of the LLM framework. More detailed results can be found in Supplementary (Tables S3 and S4).

At WCM, our two-stage LLM framework with three-shot in-context learning (ICL) using Gemini 2.5 Flash achieved an AUC of 0.746 (Table 2, Fig. 2). This configuration significantly outperformed the strongest traditional ML baseline, CatBoost trained on structured features (AUC 0.723; DeLong's Z = 2.013, p = 0.044, 95% CI [0.001, 0.045]), and substantially exceeded logistic regression (AUC 0.691; Z = 3.674, p < 0.001, 95% CI [0.025, 0.085]). The ZS-Disposition baseline adapted from Williams et al.^10^, which predicts admission rather than RVA via prompt-based inference without retrieval, achieved AUC 0.672 (Table 2), confirming that prompts targeting the more general task of admission do not substitute for RVA-specific prediction.

In-context learning provided clear and stable gains at WCM. Even one example per class meaningfully improved performance over zero-shot prompting (Gemini ICL = 1 AUC 0.733 vs. ICL = 0 AUC 0.703; Table 3); the three-shot configuration achieved AUC 0.746, a > 2% improvement over the strongest baseline (Fig. 2, left). Qualitatively, the ROC curves for zero-shot and ICL configurations overlapped from the origin to a false-positive rate of approximately 0.3, indicating comparable performance on lower-acuity, high-confidence cases. ICĽs advantage emerged in the more ambiguous middle range, where confidence is lower and additional context from examples helps the LLM refine borderline predictions. With Qwen3–8B as the backbone, the same qualitative pattern held but at lower overall AUC (best 0.713 at three-shot; Table 2) and with less stable ICL gains across the full sweep (Table 3, Fig. 2 right), indicating that backbone scale and model provenance influence both severity assessment quality and learning-from-examples dynamics.

**Figure 3:**
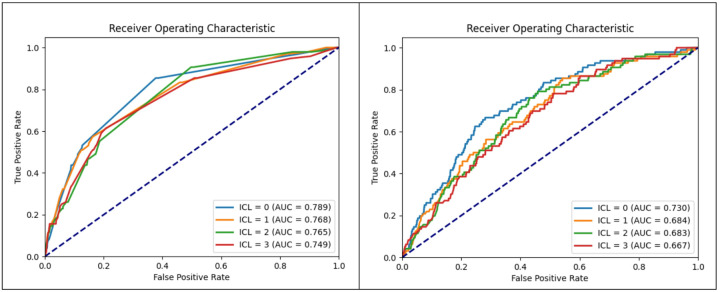
Performance of GPT-5 mini (left) and Qwen3-8B (right) on VUMC data.

At VUMC, the LLM-based pipeline did not exceed traditional ML. The best LLM configuration, GPT-5 mini with structured data plus severity summary, zero-shot, achieved AUC 0.789, but logistic regression on structured data alone reached AUC 0.799 (Table 2), a non-significant difference (Z = − 0.865, p = 0.389, 95% CI [− 0.176, 0.068]). The same pattern held against CatBoost (AUC 0.720; Z = 0.505, p = 0.679, 95% CI [− 0.095, 0.196]). Qwen3–8B at VUMC similarly did not improve significantly over either logistic regression (best AUC 0.730 vs. 0.799; Z = − 1.70, p = 0.089, 95% CI [− 0.26, 0.019]) or CatBoost (Z = − 0.187, p = 0.852, 95% CI [− 0.187, 0.154]). Across the broader sweep of traditional ML configurations at VUMC, AUCs ranged from 0.635 (DICE) to 0.799 (LR; Table 3), with most exceeding their WCM counterparts. Unlike at WCM, additional ICL examples did not consistently improve performance for either GPT-5 mini or Qwen3–8B at VUMC (Table 3, Fig. 3), suggesting that the value of incontext exemplars is itself site-conditional.

Cross-site comparison. The gap in ZS-Disposition AUC between sites (WCM Gemini 0.672 vs. VUMC GPT-5 mini 0.610; Table 2) confirms that prompts targeting admission, rather than RVA specifically, generalize poorly to RVA prediction, motivating the specialized two-stage framework evaluated above. More substantively, the LLM-over-ML margin observed at WCM did not replicate at VUMC, even as absolute discrimination at VUMC was higher for both approaches. This pattern is consistent with VUMC's structured EHR data capturing more of the dominant signal for RVA risk, leaving a smaller marginal value for the LLM's contribution from narrative text. The ablation evidence supporting this interpretation is presented in the following Data requirements section.

### Data requirements

Beyond discrimination, the pipeline that achieves the WCM gain has specific input requirements. We assessed these by ablating the contribution of (i) structured EHR variables, (ii) clinical narrative notes, and (iii) the LLM-generated severity summary, both within traditional embedding-based ML pipelines using contrastive learning and within the LLM-prediction pipeline.

Structured EHR alone is a strong but bounded baseline. Traditional ML models trained on structured variables (demographics, medications, conditions, observations, procedures) achieved AUC 0.691–0.723 at WCM and 0.720–0.799 at VUMC (Table 2; logistic regression and CatBoost). Notably, structured-only AUC was substantially higher at VUMC than at WCM, indicating that VUMC's structured EHR captures more of the dominant signal for RVA risk than does WCM's. Contrastive-learning analysis in the embedding space confirmed that RVA and non-RVA cases derived from structured data alone are not cleanly separable at either site (Supplementary Figs. S1 and S2), suggesting that structured-only discriminability is bounded by features that may not become separable even with supervised representation learning.

Adding raw clinical notes via traditional text embeddings produces inconsistent and bounded effects across sites. At WCM, embedding-based fusion degraded structured-only CatBoost performance (AUC fell from 0.723 to 0.666; Tables 2 and 3). At VUMC, the same approach modestly improved CatBoost (from 0.720 to 0.765; Tables 2 and 3) but did not exceed the structured-data logistic regression baseline (AUC 0.799). Naive embedding-based fusion therefore neither consistently recovers the predictive signal in narrative documentation, nor closes the gap to a simple, well-fitted structured-data model when that model already discriminates strongly.

At VUMC, LLM-generated severity summaries do not rescue embedding-based ML and perform worse than raw-note embeddings. Substituting raw notes with the LLM-generated severity summary (and re-embedding) produced AUCs of 0.720 (Gemini) and 0.712 (Qwen) at WCM, and 0.677 (GPT-5 mini) and 0.656 (Qwen) at VUMC (Table 3), at or below the structured-only CatBoost baseline at each site, and at VUMC also below the embedding-plus-raw-notes configuration. Severity summaries therefore do not contribute predictive value when treated as additional embedded features for a downstream classifier. However, severity summaries do contribute predictive value when consumed by the LLM as natural-language context. When the same severity summaries are returned to an LLM that performs the prediction directly, AUC rises from 0.703 (Gemini ICL = 0) to 0.746 (Gemini ICL = 3) at WCM, and reaches 0.789 (GPT-5 mini ICL = 0) at VUMC (Tables 2 and 3). This contrast suggests that severity summaries add discriminative value only when consumed by the LLM as natural-language context, not when treated as embedded features for a downstream classifier in the configuration tested here.

In terms of implication for deployment, reproducing the WCM gain requires (i) access to structured EHR variables, (ii) access to clinical narrative documentation, and (iii) an LLM-based prediction step capable of in-context reasoning over both, since severity summaries add discriminative value only when consumed by the LLM as natural-language context rather than as embedded features. Where structured data already strongly discriminates outcomes, as at VUMC, the LLM pipeline may not add value over simpler ML baselines.

### Hallucination risk

Using a FActScore-inspired approach,^48^ Gemini 2.5 Flash had a slightly higher burden of unsupported asserted facts than Qwen3–8B. Across ICL settings, around 24.2% of Gemini summaries contained at least one detected unsupported asserted clinical fact, with a mean of 0.309 unsupported facts per summary, compared with around 21.7% and 0.277 per summary for Qwen3-8B. Most detected unsupported facts were conditions, with Gemini showing a higher mean unsupported condition rate than Qwen3-8B (0.274 vs. 0.231 per summary). These results confirm that more capable LLMs may generate more clinically specific summaries which can improve prediction performance and interpretability, but also increase the chance of unsupported asserted details.

### Operational cost

Real-world deployment of an RVA prediction pipeline at ED scale must account for per-encounter resource consumption and inference latency. We profiled token use, GPU-hours, and latency across the four backbone by site configurations evaluated above and contrasted them against traditional ML baselines (Table 4). The two-stage pipeline requires two sequential model calls per encounter, severity summary generation and RVA prediction; hence, reported token consumption reflects the sum of both calls.

Commercial LLM inference. At WCM, Gemini 2.5 Flash consumed approximately 1,051, 1,291, 1,529, 1,763 million tokens for experiments with ICL = 0, 1, 2, 3 respectively (5,634 million tokens in total) across the 100,000-encounter cohort, averaging approximately 10,511, 12,906, 15,292, 17,632 tokens per encounter (input plus output, two calls combined) for experiments with ICL = 0, 1, 2, 3 respectively (56,340 in total), at mean inference latency 1.22, 1.29, 1.36, 1.44 seconds per encounter for experiments with ICL = 0, 1, 2, 3 respectively. At VUMC, GPT-5 mini consumed approximately 68.4, 92.6, 116.4, and 140.0 million tokens across the 10,000-encounter cohort for ICL = 0,1,2, and 3, respectively, averaging approximately 7,599, 10,284, 12,930, and 15,554 tokens per encounter, at mean latency 1.39, 1.68, 2.02, 2.09 seconds, respectively. Projected to 100,000 encounters per year—a typical high-volume academic ED—commercial inference at the levels observed here would consume approximately 5,634 million tokens annually at WCM and 4,637 million tokens annually at VUMC.

Open-source LLM inference (Qwen3–8B). Token consumption per encounter is broadly comparable to the commercial backbones since the same two-stage prompts are used, with differences arising from tokenizer-specific encoding (e.g., Qwen3–8B consumed ~ 23% fewer tokens than Gemini 2.5 Flash at WCM for identical prompt content). WCM ran the full 100,000-encounter evaluation on a single NVIDIA RTX 4090 in approximately 69, 71, 80, and 92 GPU-hours and 713, 956, 1,198, and 1,437 million tokens (4,304 million tokens in total) for experiments with ICL = 0, 1, 2, and 3 respectively; VUMC ran on a single NVIDIA A6000 in approximately 5.31, 5.70, 6.96, 8.28 GPU-hours. Mean inference latency for Qwen3–8B was 2.76, 2.85, 3.20, 3.68 seconds per encounter for experiments with ICL = 0, 1, 2, and 3 respectively at WCM and 2.09, 2.24, 2.74, and 3.26 seconds at VUMC. These measurements exclude one-time hardware acquisition, ongoing maintenance, and ML-operations overhead, which fall on the deploying institution rather than on a per-call basis.

Traditional ML baselines. Logistic regression and CatBoost trained and inferred on CPU in under 3 minutes per site end-to-end at both sites, with inference latency on the order of 1 milliseconds per encounter and no marginal compute cost beyond standard EHR analytics infrastructure already present in most academic medical centres.

Resource-versus-discrimination trade-off at each site. At WCM, the commercial LLM pipeline gained an absolute AUC of 0.023 over the strongest ML baseline (CatBoost 0.723 → Gemini ICL = 3 0.746) at a budget of approximately 56,300 tokens and over 1 second of latency per encounter. At VUMC, the commercial LLM pipeline did not exceed the logistic-regression baseline (see Discrimination performance), and the same token and latency budget therefore pays for parity rather than improvement. Open-source Qwen3–8B avoids per-call API consumption but shifts resource demand toward dedicated GPU infrastructure and ML-operations capacity—a trade-off that depends on each site's ability to maintain local model serving over a multi-year deployment horizon.

### Subgroup analysis

In analyses stratified by Elixhauser Comorbidity Index (Table 5), Gemini 2.5 Flash with in-context learning showed the strongest discrimination across both comorbidity strata. For encounters with ECI ≤ 0, Gemini improved from an AUROC of 0.674 without ICL to 0.727 with three ICL examples, exceeding CatBoost (0.709) and logistic regression (0.661). For encounters with ECI > 0, Gemini similarly improved from 0.680 to 0.701 across ICL = 0 to ICL = 3, again outperforming CatBoost (0.671) and logistic regression (0.663). In contrast, Qwen3–8B showed weaker performance in both strata, with AUROCs ranging from 0.655 to 0.705 for ECI ≤ 0 and 0.641 to 0.657 for ECI > 0. These findings indicate that Gemini's advantage over traditional ML was preserved across both lower and higher comorbidity-burden encounters, with Gemini ICL = 3 consistently performing best. The improvement was modest but directionally consistent, indicating that the LLM's discrimination was not limited to patients with greater coded comorbidity burden.

### Disagreement analysis

On the common test set of Gemini ICL = 3 and CatBoost, which included 18,011 encounters and 231 RVA cases, Gemini ICL = 3 achieved an AUROC of 0.743, while CatBoost achieved an AUROC of 0.724. Among the 231 RVA cases, both models correctly identified 134 cases (58.01%), CatBoost missed but Gemini caught 30 cases (12.99%), Gemini missed but CatBoost caught 38 cases (16.45%), and both models missed 29 cases (12.55%). For those RVA cases that CatBoost missed but Gemini caught, the most common conditions were essential hypertension (30.00%), alcohol intoxication (16.67%), hyperlipidemia (16.67%), dizziness and giddiness (13.33%), electrocardiogram abnormality (13.33%), nausea and vomiting (13.33%), and vomiting (13.33%); for those RVA cases that Gemini missed but CatBoost caught, the most common conditions were essential hypertension (34.21%), hyperlipidemia (34.21%), abdominal pain (18.42%), chest pain (18.42%), dyspnea (18.42%), gastroesophageal reflux disease without esophagitis (18.42%), and anxiety disorder (15.79%). The CatBoost-missed/Gemini-caught cases were most often assigned moderate acuity by the LLM (53.33%), followed by low acuity (26.67%) and critical acuity (20.00%), whereas the Gemini-missed/CatBoost-caught cases were predominantly low acuity (65.79%), followed by moderate acuity (31.58%) and critical acuity (2.63%). The CatBoost-missed/Gemini-caught group was 60.00% male and 40.00% female, with race recorded most commonly as White (36.67%), Black or African American (30.00%), or other combinations not described (16.67%), and ethnicity recorded as not Hispanic or Latino in 83.33% and Hispanic or Latino in 16.67%. The Gemini-missed/CatBoost-caught group was 55.26% male and 44.74% female, with race recorded most commonly as Black or African American (36.84%), White (36.84%), other combinations not described (10.53%), or Asian (10.53%), and ethnicity recorded as not Hispanic or Latino in 86.84% and Hispanic or Latino in 13.16%. These disagreement patterns suggest that Gemini added value for a small subset of RVA cases with higher acuity signals and several symptom- or presentation-oriented conditions, whereas CatBoost more often caught lower-acuity RVA cases with common chronic comorbidity patterns. However, the small size of each disagreement group means these subgroup patterns should be interpreted as descriptive rather than definitive evidence of systematic model behavior.

## Discussion

This study evaluated a two-stage LLM-based framework for RVA prediction across two independent academic health systems through a real-world feasibility lens, examining the conditions under which an LLM-based pipeline outperforms traditional ML— and the costs of that gain when it occurs. Across four feasibility axes (discrimination, data requirements, operational cost, and hallucination risk), the case for LLM deployment proves site-conditional rather than universal.

At WCM, CatBoost trained on structured data remains a strong baseline, achieving an AUC of 0.723 with 80% of the training set. However, our LLM-based method using only 10% of the data as a retrieval corpus exceeds this benchmark: the three-shot configuration reaches an AUC of 0.746, offering a clinically meaningful > 2% improvement given the rarity of RVA events and the operational importance of early identification. External validation at VUMC reframes rather than confirms generalisability. Both LLM and ML approaches achieved higher absolute discrimination at VUMC than at WCM, indicating that both translate to a distinct patient population. However, the LLM-over-ML advantage observed at WCM did not hold: logistic regression on structured data alone (AUC 0.799) matched the best LLM configuration (GPT-5 mini AUC 0.789, n.s.). This pattern is consistent with VUMC's structured EHR data already capturing the dominant signal for RVA risk, leaving less marginal value for the LLM's contribution from narrative text. The deployment decision is therefore site-conditional: an LLM-based pipeline adds value where structured data underspecifies risk, and offers parity—at higher cost—where structured data is already strongly predictive.

At WCM, the performance gains from ICL were most pronounced for clinically ambiguous cases, as evidenced by the divergence of ROC curves at lower specificity thresholds. This suggests that in-context exemplars help the LLM refine risk estimation precisely where structured signals are insufficient, a finding with important clinical implications, since borderline cases represent the highest-stakes disposition decisions in the ED. The principal contribution of this work is therefore conceptual as much as incremental: by instructing the LLM to generate a clinically interpretable severity abstraction prior to outcome prediction, we approximate the kind of cognitive synthesis clinicians perform at the point of discharge, rather than merely adding textual embeddings as additional features. The ablation findings support this framing, where raw notes alone underperform severity-summarized inputs, indicating that structured abstraction of clinical context, rather than raw text representation, is the operative mechanism driving the signal.

At WCM, even a single exemplar per class substantially improved discrimination relative to zero-shot prompting (Gemini ICL = 1 AUC 0.733 vs. ICL = 0 AUC 0.703). ROC curves for zero-shot and ICL configurations overlapped at low false-positive rates but diverged at higher false-positive rates (Fig. 2), consistent with ICL providing greater discrimination in the more ambiguous middle range of the prediction distribution. This pattern suggests that exemplars help the LLM refine risk estimation precisely where structured signals are insufficient, a clinically important property, since borderline cases represent the highest-stakes disposition decisions in the ED. At VUMC, however, neither GPT-5 mini nor Qwen3-8B showed consistent improvement with additional exemplars, indicating that the benefit of ICL is itself site-conditional and depends on patient population and clinical documentation practices. At sites where ICL does help, performance gains taper beyond two exemplars (Table 3, Fig. 2), suggesting that small retrieval corpora suffice — an important consideration for deployment at expected ED throughput.

The contrast, severity summaries help when consumed by the LLM but not when consumed as embedded features, suggests that the LLM's natural-language reasoning over the summary, not the summary content alone, is the operative mechanism. We hypothesise that the LLM uses contextual elements documented in narrative notes: symptom trajectory, clinician gestalt, and response to therapy, to refine borderline predictions, consistent with clinical practice in which physicians rely heavily on narrative documentation to identify patients at elevated risk.^26^ Direct content analysis of severity summaries, examining the extent to which they synthesise these elements, is a planned follow-up.

Beyond the local mechanism by which severity summaries add value, the cross-site contrast at VUMC suggests a more general deployment principle: where structured EHR data already captures the dominant predictive signal, the LLM's narrative-derived contribution shrinks toward zero. The value of LLM-based abstraction appears tied to how much of the predictive signal is already captured by structured EHR variables at a given site. Site-specific calibration or local adaptation will likely remain necessary for reliable deployment across diverse clinical settings^42,49–51^.

Three threads of prior work directly inform this study, and each is extended in a different direction. First, Henriksson et al. demonstrated, in a six-ED multimodal fine-tuning study, that combining structured and unstructured data outperforms unimodal models for COVID-19 outcome prediction including readmission;^36^ we extend this to general ED RVA prediction with frozen commercial-grade LLMs and in-context learning, and document a site-conditional advantage that has not previously been surfaced in multi-site clinical-prediction work. Second, Williams et al. evaluated ChatGPT on ED disposition recommendations and found that GPT-4 could not yet outperform human physicians; we extend this to a head-to-head comparison against locally-trained ML, finding that the comparison is itself site-conditional rather than a property of the model.^10^ Third, Brown et al. systematically benchmarked GPT-3.5 and GPT-4 on clinical prediction tasks and reported weaker LLM performance across discrimination, calibration, fairness, and robustness; with newer commercial models and a structured two-stage prompting strategy, we observe LLM discrimination much closer to and in select cases exceeding traditional ML, suggesting that the gap reported in earlier benchmarks may reflect both model evolution and prompting strategy.^11^

Beyond predictive performance, our long-term aim is to deploy this framework in real-world ED settings to support physicians’ decision-making and improve operational efficiency. Deploying an RVA prediction framework, whether LLM- or ML-based, requires translating discrimination performance into operational thresholds aligned with local capacity and risk tolerance. Rather than a single statistical optimum, sites could use the model's probabilistic output to set tiered (low / medium / high) risk bands reflecting the relative costs of false positives and false negatives in their workflow. False positives consume scarce ED and inpatient capacity through unnecessary monitoring or admissions; false negatives represent missed opportunities for proactive follow-up. Low sensitivity is a persistent challenge for any model trained on highly imbalanced data because RVA prevalence is approximately 1%; however, balanced few-shot demonstrations allowed LLMs to maintain competitive sensitivity at WCM, where the LLM pipeline had a discrimination advantage. Where ML achieves comparable AUC at lower cost (as at VUMC), the same threshold logic applies to the simpler model, with no operational penalty for foregoing the LLM.

Our study has several limitations. The first limitation is the lack of compatible LLMs across sites, due to factors such as enterprise purchasing decisions and governance that are beyond researchers’ control. Evaluation relied on retrospective data from academic medical centres; validation in community EDs and prospective deployment workflows remain untested. RVA ascertainment was restricted to return visits within each health system; patients who returned to unaffiliated emergency departments would not have been captured, likely underestimating the true RVA rate and introducing ascertainment bias^52^. We currently rely only on ground-truth RVA labels for ICL and lack exemplar demonstrations for the severity assessment agent, which the ablation study suggests is important. The site-conditional pattern observed here is consistent with, but does not formally demonstrate, a hypothesis that the predictive value of LLM-based abstraction depends on the relative informativeness of structured versus narrative EHR data. Direct measurement (e.g., LLM evaluation on structured input alone, or feature-importance decomposition of a joint model) is left to future work.

Logistic regression was evaluated only on structured EHR data; CatBoost served as the default traditional-ML representative for embedding-based and severity-augmented variants because of its better-known performance on high-dimensional tabular inputs. The VUMC sample contained only 106 RVA cases, yielding wider confidence intervals on between-model comparisons; the non-significant differences reported at VUMC are therefore consistent with both true equivalence and underpowered detection of small differences. Beyond discrimination, calibration, and robustness to documentation perturbation were not evaluated and are next-priority safety dimensions for this pipeline^11,49,51^. Finally, this work did not explore reasoning over RVA causes; future work will consider direct causal reasoning over RVA-precipitating factors.

Hallucination assessment used a rule-based faithfulness metric inspired by FActScore, and was conducted at WCM only; corresponding rates for VUMC were not generated. The metric represents a conservative screening signal rather than a definitive hallucination rate, since it does not capture narrative confabulation, inappropriate severity assignment, or asserted facts that fall outside the curated structured-concept ontology. LLM-as-judge adjudication or manual chart review by emergency physicians would provide ground-truth validation; both, along with cross-site replication, should be follow-up work.

In a multi-site evaluation of LLM-based clinical prediction for ED RVA, we find that the case for deployment is conditional rather than universal. At WCM, an LLM-based pipeline outperformed the strongest traditional ML baseline; at VUMC, structured-data logistic regression matched the LLM at substantially lower operational cost. The deciding factor appears not to be LLM capability but rather the relative informativeness of structured versus narrative EHR data at each site, a property reflected in the substantial difference in structured-only baseline AUC between WCM (0.723) and VUMC (0.799). Practitioners considering LLM deployment for clinical prediction should evaluate site-specific structured-only baselines, quantify per-encounter operational cost at expected throughput, and characterise hallucination risk in any LLM-generated artefacts before committing.

## Methods

### Ethics approval

This single-IRB study was approved by Biomedical Research Alliance of New York (BRANY): Study ID# 23-08-620-1550. Within each site, this study was approved by the WCM IRB (23-08026440) and VUMC IRB (242010 and 241171). All research was conducted in accordance with the Declaration of Helsinki and relevant institutional guidelines. Given the retrospective design, the requirement for informed consent was waived.

#### Data

We used electronic health record (EHR) data from four large, urban, EDs from two academic medical centers participating in an observational medical outcomes partnership (OMOP) common data model instance during 2022–2025. All EDs contributed structured and unstructured data, including demographics, conditions, medications, procedures, observations and clinical notes, with all computation performed in a HIPAA-compliant environment. To mimic real-world clinical deployment and avoid information leakage, we excluded discharge instructions and included only notes generated during the ED encounter; all note classes can be found in the Supplementary section (Tables S1 and S2). Structured variables included demographics (age, sex, race/ethnicity), conditions, medications ordered during the ED visit, measurements (laboratory and vital signs), observations and any procedures performed. To understand the model’s performance across patient population, we also did a subgroup analysis by Elixhauser comorbidity index.^53^

The primary task is to use an LLM to predict the probability that an index ED visit would result in a 9-day RVA. Motivated by prior ED work showing the importance of severity assessment for downstream risk prediction,^7,10^ we implemented a two-stage architecture. In the first stage, an LLM generated structured severity summaries by jointly processing free-text ED documentation (e.g. ED notes and triage notes) alongside structured EHR variables comprising demographics (e.g., age and gender), medications, procedures (diagnostic or therapeutic actions performed by a clinician), conditions (diagnoses), measurements and observations (e.g., lifestyle, social history, and family history). The resulting severity summaries capture clinically salient markers of patient acuity by explicitly distinguishing unstructured indicators, including symptom trajectory, response to therapy, and clinician gestalt documented in narrative notes, from structured indicators such as vital sign instability and abnormal laboratory values. Prompts for generating severity summaries were iteratively refined with study emergency physicians to ensure clinical validity. The acuity summarization step also reduces long clinical notes into a format that fits within LLM token limits and substantially accelerates inference.

**Figure 4:**
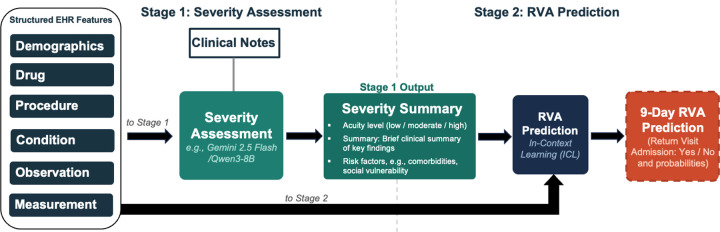
Two-stage LLM-based framework for ED RVA prediction

In the second stage, the summary and the structured variables are returned to an LLM that performs binary RVA prediction via zero-shot or in-context learning (ICL), with probabilities derived from yes/no token log-probabilities (Fig. 4). For each target case, a deterministic retriever^54^ compared the structured variables of the index visit to those in the retrieval corpus. Similarity was computed as the count of exact matches across demographics, conditions, medications, observations, and procedures. We chose this approach for its computational efficiency, avoiding the overhead of embedding generation while capturing patient characteristics. The top-*k* similar encounters were incorporated into the prompt as in-context exemplars, each including structured data, its severity summary, and its known RVA outcome. Following prior work^55,56^ on clinical LLM retrieval and mimicking a real-world deployment scenario, we first sorted the dataset by visit date and selected the earliest 10% of RVA cases and the earliest 10% of non-RVA cases, yielding a retrieval corpus containing 10% of the overall cohort. Each exemplar included the full structured variable set and the LLM-generated severity summary. The most recent remaining 90% of data were used for evaluation.

**Figure 5:**
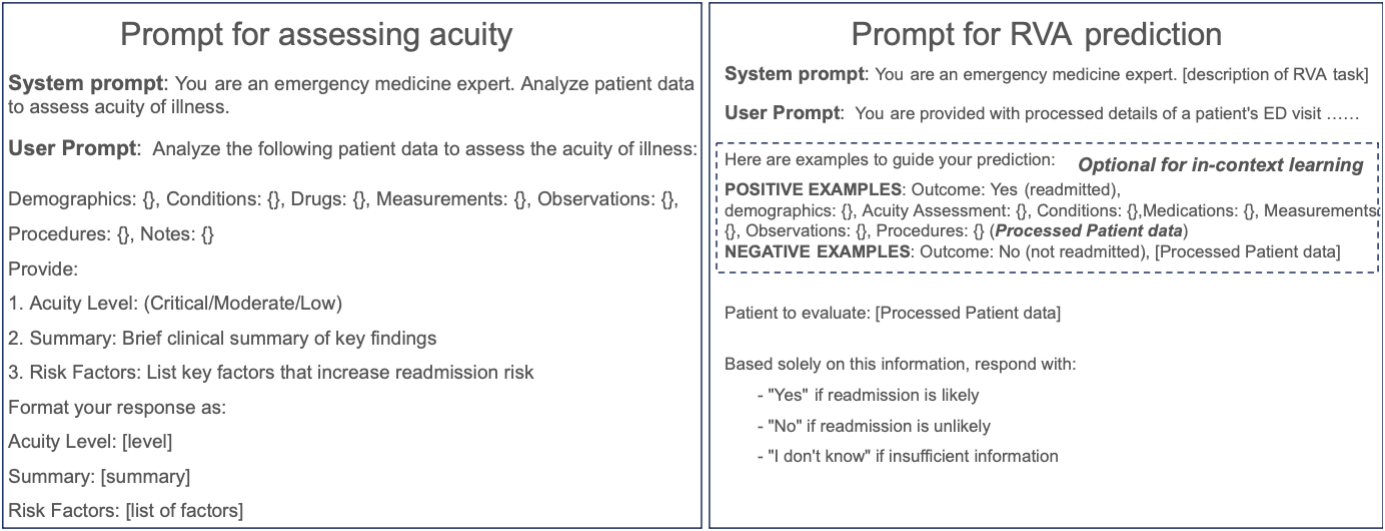
Prompt structure

The index case’s structured variables and severity summary were then appended to a task-specific prediction prompt. Prompts (Fig. 5) were iteratively refined with ED physicians to ensure unambiguous interpretation of “return visit admission” and to reduce ambiguity in borderline scenarios. Full prompts are provided in the Supplementary. The final prompt instructed the LLM to output a binary prediction (“yes” or “no”), using temperature = 0 to ensure deterministic responses. Temperature is a critical parameter controlling the randomness of LLM generation, with higher values producing more diverse and potentially more hallucinated outputs. To derive probability estimates, we extracted the log-probabilities assigned to the “yes” and “no” tokens and applied a softmax transformation. This approach follows established methods for estimating calibrated probabilities from generative LLMs.^47^

#### Baselines and ablation studies

We evaluated the two-stage LLM-based framework against three categories of baseline approaches. The first category comprised traditional ML models trained on structured EHR variables: logistic regression, CatBoost^57^, and deep significance clustering (DICE^58^). The second category comprised embedding-based ML models trained on dense representations derived from structured features and clinical notes. The third category was a zero-shot LLM baseline adapted from Williams et al.^10^ (hereafter “ZS-Disposition”), which predicts ED disposition rather than RVA via prompt-based inference without retrieval; because patients admitted during the index visit are by definition ineligible for RVA, admission prediction served as a clinically grounded proxy outcome for RVA identification.

For traditional ML models, structured inputs underwent standard preprocessing: one-hot encoding for categorical variables and min-max normalisation for continuous variables. For LLM-based methods (including the proposed two-stage framework and the ZS-Disposition baseline), raw OMOP codes were replaced with their natural-language descriptions (e.g., condition names, procedure names) to expose the model to clinically meaningful semantics and leverage LLM pretraining knowledge. For embedding-based models, we generated text embeddings using either raw clinical notes or LLM-generated severity summaries—each combined with the structured EHR variables—to isolate the marginal contribution of the severity assessment stage relative to raw-text input. To evaluate whether supervised optimisation of the embedding space could improve discrimination, we additionally applied contrastive learning to the text embeddings generated by bert-base-uncased^59^: paired examples from the training set were used to optimise a contrastive loss that maps cases sharing the same RVA label closer together in embedding space while pushing apart those with different labels. The resulting representations were projected to two-dimensional space for visualisation (Supplementary Figs. S1 and S2) and used as input features for downstream classifiers.

We characterised the contribution of in-context learning by sweeping configurations from zero-shot through three exemplars per class (ICL = 0, 1, 2, 3) for both backbones at both sites. We characterised the effect of backbone model scale and provenance by evaluating a small open-source model (Qwen3-8B) at both sites alongside larger commercial models. Model availability differed by institution due to local HIPAA-compliant deployment constraints: Gemini 2.5 Flash was used at WCM and GPT-5 mini at VUMC. Qwen3-8B served as the cross-site open-source comparator, enabling a like-for-like comparison across sites that the differing commercial deployments do not permit.

Finally, to characterise model behaviour across patient strata, we conducted a subgroup analysis by Elixhauser Comorbidity Index (ECI)53, a validated summary measure of comorbidity burden derived from administrative diagnosis codes. Encounters were stratified into two groups (ECI ≤ 0 and ECI > 0), and AUROC was computed within each stratum for each model configuration (Table 5). Findings from all ablations are presented in Results.

#### Hallucination measurement

We also evaluated whether LLM-generated severity assessment contained unsupported clinical facts using a stricter rule-based hallucination metric based on FActScore.^48^ In this analysis, each severity assessment was screened for asserted patient-specific clinical facts involving conditions, medications, measurements, and procedures. A fact was counted as unsupported only if it was asserted in the generated severity assessment but could not be matched to the structured EHR input or note summary for that encounter. To reduce false positives, we excluded negated, hypothetical, risk-only, differential-diagnosis, family-history, and refused-procedure, and we allowed common clinical inferences such as anemia from low hemoglobin, hypertension from elevated blood pressure, and pyuria from urinalysis findings.

#### Cost and latency measurement

For each site × backbone configuration, we logged per-encounter input and output token counts at the API call site for commercial models (Gemini 2.5 Flash at WCM, GPT-5 mini at VUMC). For the open-source Qwen3–8B model, we recorded GPU wall-clock time on local inference hardware (NVIDIA RTX 4090 at WCM, NVIDIA A6000 at VUMC). Per-encounter inference latency was logged at both sites. Token consumption is reported as mean and total per site, plus a projected per-100,000-encounters figure to support deployment-decision contexts where ED throughput is high. Traditional ML baselines were trained and evaluated on CPU; their compute cost is treated as negligible relative to LLM inference and reported as such.

#### Statistical Methods

Between-group differences in patient demographics were assessed using Welch’s t-test for continuous variables (age) and Pearson’s chi-squared test for categorical variables (sex, race, and ethnicity), with p-values reported in Table 1. Model discrimination was evaluated using the area under the receiver operating characteristic curve (AUROC), which served as the primary performance metric given class imbalance and precedent in ED risk-stratification research. AUROC differences were measured using DeLong’s test. Sensitivity, specificity, and positive likelihood ratio were reported as secondary metrics. For all traditional machine learning classifiers, the optimal classification threshold was selected using Youden’s J statistic on the evaluation set. All analyses were conducted in Python (version 3.10). All commercial model inference was conducted within HIPAA-compliant, PHI-approved hosting environments at each respective institution.

## Supplementary Material

Supplementary Files

This is a list of supplementary files associated with this preprint. Click to download.

- supplementaryv2.pdf

## Figures and Tables

**Table 1:** Patient demographics at WCM and VUMC

Variable	Value	WCM				VUMC			
		RVA	Non-RVA	All	P-value	RVA	Non-RVA	All	P-value
Numerical Variables					Welch's T-Test p-value				Welch's T-Test p-value
Age (sd)		54.4 (19.1)	47.8 (19.1)	47.9 (19.1)	<0.0001	53.6 (20.1)	44.2 (18.3)	44.4 (18.3)	<0.0001
Categorical Variables					Pearson's Chi-squared test p-value				Pearson's Chi-squared test p-value
Gender					<0.0001				0.428
	Female	604 (48.5%)	54107 (54.8%)	54710 (54.7%)		54 (50.9%)	5468 (55.3%)	5522 (55.2%)	
	Male	643 (51.5%)	44638 (45.2%)	45282 (45.3%)		52 (49.1%)	4426 (44.7%)	4478 (44.8%)	
	Nonbinary	0 (0.0%)	8 (0.0%)	8 (0.0%)		0 (0.0%)	0 (0.0%)	0 (0.0%)	
Race					<0.0001				0.025
	White	379 (30.4%)	29354 (29.7%)	29733 (29.7%)		68 (64.2%)	5859 (59.2%)	5927 (59.3%)	
	Black or African American	425 (34.1%)	28696 (29.1%)	29121 (29.1%)		9 (8.5%)	1217 (12.3%)	1226 (12.3%)	
	Others	305 (24.5%)	29812 (30.2%)	30117 (30.1%)		3 (2.8%)	73 (0.8%)	76 (0.8%)	
	Asian	119 (9.5%)	9915 (10.0%)	10034 (10.0%)		0 (0.0%)	96 (1.0%)	96 (1.0%)	
	Unknown/Declined	19 (1.5%)	976 (1.0%)	995 (1.0%)		*26* *(24.5%)*	*2649* *(26.8%)*	*2675* *(26.8%)*	
Ethnicity					<0.0001				0.439
	Not Hispanic or Latino	923 (74.0%)	70273 (71.2%)	71196 (71.2%)		38 (35.8%)	3008 (30.4%)	3046 (30.5%)	
	Hispanic or Latino	315 (25.3%)	28320 (28.7%)	28635 (28.6%)		7 (6.6%)	829 (8.4%)	836 (8.4%)	
	Unknown	9 (0.7%)	158 (0.2%)	167 (0.2%)		61 (57.5%)	6057 (61.2%)	6118 (61.2%)	
Record num (sd)									
	All	136.3 (71.8)	108.8 (66.6)	109.2 (66.8)	<0.0001	124.1 (56.8)	112.8 (62.4)	113.0 (62.4)	0.045752
	Notes	7.3 (7.3)	6.4 (6.9)	6.4 (6.9)	<0.0001	6.1 (3.2)	5.7 (3.0)	5.7 (3.0)	0.180548
	Structured	128.9 (68.6)	102.4 (63.8)	102.7 (63.9)	<0.0001	117.9 (55.6)	107.1 (60.8)	107.2 (60.8)	0.049598

**Table 2:** Discrimination performance

Approach	Configuration	WCM AUC	VUMC AUC
**Traditional ML (structured EHR only)**			
**Logistic regression**	**80% training**	**0.691**	**0.799**
CatBoost	80% training	0.723	0.720
Two-stage LLM (severity summary + ICL, best config)			
Commercial backbone	Gemini 2.5 Flash, ICL = 3 (WCM) / GPT-5 mini, ICL = 0 (VUMC)	0.746	0.789
Open-source backbone	Qwen3–8B, best ICL config	0.713 (ICL = 3)	0.730 (ICL = 0)
Naive prompt baseline (ZS-Disposition, no severity, no ICL)			
Commercial backbone	Gemini (WCM) / GPT-5 mini (VUMC)	0.672	0.610
Open-source	Qwen3–8B	0.662	0.643

* Baseline: ZS-Disposition baseline

**Table 3:** Ablation studies

Method	Input	WCM AUC	VUMC AUC
**Two-stage LLM, ICL sweep (commercial backbone)**	**structured + severity summary**		
**Gemini 2.5 Flash (WCM) / GPT-5 mini (VUMC), ICL = 0**		**0.703**	**0.789**
ICL = 1		0.733	0.769
ICL = 2		0.738	0.769
ICL = 3		0.746	0.758
Two-stage LLM, ICL sweep (Qwen3–8B)	structured + severity summary		
ICL = 0		0.683	0.730
ICL = 1		0.712	0.684
ICL = 2		0.706	0.684
ICL = 3		0.713	0.667
Embedding-based ML (CatBoost)			
Structured + raw notes (text embeddings)		0.666	0.765
Structured + severity summary (commercial-LLM-generated, embedded)		0.720 (Gemini)	0.677 (GPT-5 mini)
Structured + severity summary (Qwen-generated, embedded)		0.712	0.656
Deep neural network			
DICE	structured EHR	0.674	0.635

**Table 4:** Token consumption, GPU-hours, and inference latency by site and model.

Site	Approach	Backbone	Mean tokens / encounter	Total tokens (cohort)	GPU-hours	Mean latency / encounter
WCM	Commercial LLM	Gemini 2.5 Flash	~ 14,075	~ 1,409M	N/A (cloud API)	1.22–1.44 s
WCM	Open-source LLM	Qwen3–8B (NVIDIA RTX 4090)	~ 10,760	~ 1,076M	69–92	2.76–3.68 s
WCM	Traditional ML	CatBoost / Logistic regression (CPU)	N/A	N/A	N/A (CPU, < 3 min total)	Trivial
VUMC	Commercial LLM	GPT-5 mini	~ 11,600	~ 417M	N/A (cloud API)	1.39–2.09 s
VUMC	Open-source LLM	Qwen3–8B (NVIDIA A6000)	~ 11,600	~ 418M	5.3–8.3	2.09–3.26 s
VUMC	Traditional ML	CatBoost / Logistic regression (CPU)	N/A	N/A	N/A (CPU, < 1 min total)	Trivial

**Table 5:** Performance across Elixhauser Comorbidity Index (ECI)

ECI bucket	Gemini ICL0	Gemini ICL1	Gemini ICL2	Gemini ICL3	Qwen ICL0	Qwen ICL1	Qwen ICL2	Qwen ICL3	Logistic regression	CatBoost
ECI ≤ 0	0.674	0.716	0.717	**0.727**	0.655	0.705	0.693	0.703	0.661	**0.709**
ECI > 0	0.680	0.691	0.693	**0.701**	0.641	0.653	0.650	0.657	0.663	**0.671**

## Data Availability

The electronic health record data from Weill Cornell Medicine (WCM) and Vanderbilt University Medical Center (VUMC) used in this study contain protected health information (PHI) and are not publicly available due to patient privacy regulations and institutional data governance requirements. Access to these data is subject to institutional review board oversight and requires execution of appropriate data use agreements with the respective institutions. Researchers interested in accessing these data should contact the corresponding author for guidance on the institutional request process.

## Code Availability

The custom code developed for this study, including data preprocessing pipelines, LLM prompting workflows, ICL retrieval implementation, and evaluation scripts, is publicly available at https://github.com/JinsongLiu6/llm_rva_open.
